# Do Cancer and Cancer Treatments Accelerate Aging?

**DOI:** 10.1007/s11912-022-01311-2

**Published:** 2022-07-07

**Authors:** Roma Bhatia, Shernan Holtan, Najla El Jurdi, Anna Prizment, Anne Blaes

**Affiliations:** 1 Massachusetts General Hospital, Harvard Medical School, 55 Fruit Street, Boston, MA 02114 USA; 2 Division of Hematology, Oncology and Transplantation, University of Minnesota, 425 E River Pkwy, Minneapolis, MN 55455 USA

**Keywords:** Cancer, Aging, Nutrition

## Abstract

**Purpose of Review:**

This review focuses on describing the mechanisms and clinical manifestations that underlie accelerated aging associated with cancer and its treatment.

**Recent Findings:**

The direct and indirect effects of cancer and its treatment are associated with late occurrence of comorbidities that happen earlier or more frequently in cancer survivors compared to cancer-free individuals, otherwise known as accelerated aging. Use of senolytics and dietary and exercise interventions including prehabilitation, caloric restriction, and rehabilitation are currently under investigation to reverse or decelerate the aging process and will be covered in this review.

**Summary:**

Further research on how to decelerate or reverse aging changes associated with cancer and its treatment will be of paramount importance as the number of cancer survivors continues to grow.

## Introduction

Advances in detection and therapeutics have dramatically increased survival after cancer over the last quarter of the century. In fact, 70% of cancer survivors will be alive 5 or more years from diagnosis, and nearly 18% will survive 20 years or longer [1]. By 2030, it is estimated that the population of cancer survivors will exceed 22 million [1].

Despite improvements in survival, over two-thirds of all cancer survivors will have at least one chronic health condition—some because of cancer therapy itself [2]. While age is a risk factor for the development of cancer, the treatment of cancer, including chemotherapy, immunotherapy, surgery, and radiation therapy, can also accelerate biological aging processes. Furthermore, childhood cancer survivors are noted to develop twice the burden of disease by age of 45 compared to non-cancer controls [3]. On average, they are found to have seven or more chronic diseases compared to the generalized population, two of which will be seriously disabling, life-threatening, or fatal [3].

Cancer survivors are thought to develop two kinds of comorbidities: early-onset conditions, or those associated with acute effects of cancer therapy; and late-occurring conditions, or those occurring at longer-term follow-up and typically increase with age, at a faster rate compared to the generalized population [3]. Examples of these late-occurring effects include frailty [4], sarcopenia [5], cardiac dysfunction [5], and mild cognitive impairment [7].

The late occurrence of these comorbidities that happen earlier or more frequently in cancer survivors compared to cancer-free individuals is cumulatively summed up as accelerated aging [8]. The mechanisms behind accelerated aging are likely multifactorial and remain to be clearly elucidated. However, cancer and aging are thought to share several hallmarks including genomic instability, telomere attrition, and epigenetic alterations which may be implicated in accelerated aging [9]. Furthermore, treatment with cytotoxic therapy can compound these changes potentially also contributing to accelerated aging.

Herein, we aim to review the evidence to understand how and if cancer patients experience accelerated aging, the cellular mechanisms which may contribute to this phenomenon, and possible interventions to reduce the rate of or to completely reverse the phenomenon in cancer survivors.

## Hallmarks of Normal Aging

Normal aging represents the inevitable, time-dependent decline in physiologic organ function and is characterized by nine hallmarks [10]. These hallmarks are summarized in Table 1. Aggravation of these characteristics may accelerate normal aging while amelioration is thought to decelerate the aging process thereby increasing the healthy lifespan. To illustrate, exposures such as ultraviolet radiation or ionizing radiation can induce cellular DNA damage which cause cell senescence contributing to aging [11]. On the other hand, aerobic exercise can maintain genomic stability by augmenting DNA repair [12].

**Table 1:** Shared hallmarks between aging and cancer

Hallmark	Aging	Cancer & cancer therapeutics
1. Genomic instability	The general cause of aging is widely considered to be the accumulation of cellular damage. The stability of DNA is continuously under threat due to exogenous (physical, chemical, biological agents) and endogenous threats (DNA replication errors, reactive oxygen species)	Cellular damage to oncogenes and tumor suppressor genes can give rise to cancer Defects in DNA surveillance mechanisms such as DNA repair machinery can further result in genomic instability and predispose to malignant transformation
2. Telomere attrition	Accumulation of DNA damage is widespread throughout the genome; however, telomeres are particularly susceptible to age-related deterioration. Telomerase is an enzyme that augments cellular lifespan and the deficiency of which is associated with premature development of diseases due to a decrease in the regenerative capacity of tissues	Cancer cells possess high telomerase activity and are characterized by perpetuality in undergoing cell division Some chemotherapies such as cisplatin and 4-hydroperoxycyclophosphamide can directly impair telomerase in malignant and non-malignant cells
3. Epigenetic alterations	Involve age-associated changes in DNA methylation patterns, post-translational medication of histones, and chromatin remodeling	Epigenetic changes can influence cancer development in 3 ways: -Inducing mutations in genes associated with cell cycle regulation -Promoting genomic instability -Activation of proto-oncogenes and de-activation of tumor suppressor genes
4. Impaired proteostasis	Proteostasis involves mechanisms for the stabilization of correctly folded proteins and mechanisms for the degradation of proteins by proteosome or lysosome. This process is altered in aging (Koga et al. 2011)	Certain chemotherapeutic agents such as ixazomib in multiple myeloma impair protein folding and thus cause apoptosis of malignant cells
5. Deregulated nutrient sensing:	Genetic polymorphisms which reduce the function of growth hormone, insulin-like growth factor 1 (IGF-1) produced in response to GH, or downstream intracellular effectors such as AKT, mTOR, and FOXO have been linked to longevity due to being associated with lower rates of cell growth, metabolism and, hence, lower rates of cellular damage	Inhibitors of mTOR such as everolimus are used for treatment of breast cancer and renal cell carcinoma. However, use of these agents can also lead to impaired wound healing, insulin resistance, cataracts, and testicular degeneration and thus promotion of the aging phenotype
6. Cellular senescence	Defined as the stable arrest of the cell cycle. The primary purpose of the senescence is to prevent the propagation of damaged cells and trigger their demise by the immune system. Senescent cells differ in their secretory phenotype which is particularly enriched in proinflammatory cytokines and this may contribute to aging. Furthermore, the levels of p16^INK4a^ robustly correlate with chronic age in humans	Inflammatory secretome associated with senescent cells can have varying impacts on pre-malignant and malignant cells: -Either prevent pre-malignant cells from realizing their malignant potential OR -Promote a cancer conducive microenvironment The outcome of this inflammatory state depends on specific tissue, genome and/or external stimuli
7. Stem cell exhaustion	Represents the functional decline in stem cell function	Rapamycin, an inhibitor of MTOR, has been shown to promote cellular longevity, perhaps by activating genes in stem cells that can respond to oxidative stress (which otherwise are downregulated with aging and thus cause stem cell exhaustion). Rapamycin has been shown to prolong life in mice Given deregulated mTOR signaling in tumors, everolimus and other mTOR inhibitors are being studied for use in cancer treatment
8. Altered intercellular communication	Represents changes to intercellular communication including in endocrine, neuroendocrine, and neuronal pathways often described as “inflammaging,” or a proinflammatory phenotype associated with aging	Immunotherapy can stimulate intercellular communication in promoting tumor resistance and expression of this proinflammatory phenotype has been associated with aging
9. Mitochondrial dysfunction:	As cells and organisms age, the efficacy of respiratory chain is diminished and there is an increase in the accumulation of reactive oxygen species	Genetic damage leading to alterations in function of mitochondria has been associated with both cancer and aging. Mutations to mitochondrial DNA have been associated with cancer progression in various malignancies. In addition, a leading theory postulates that many cancers exhibit a depleted mitochondrial DNA content at the expense of the upregulation of genes associated with cell cycle propagation

## Shared Cellular Hallmarks Between Aging, Cancer, and Cancer Therapeutics

**Fig. 1:**
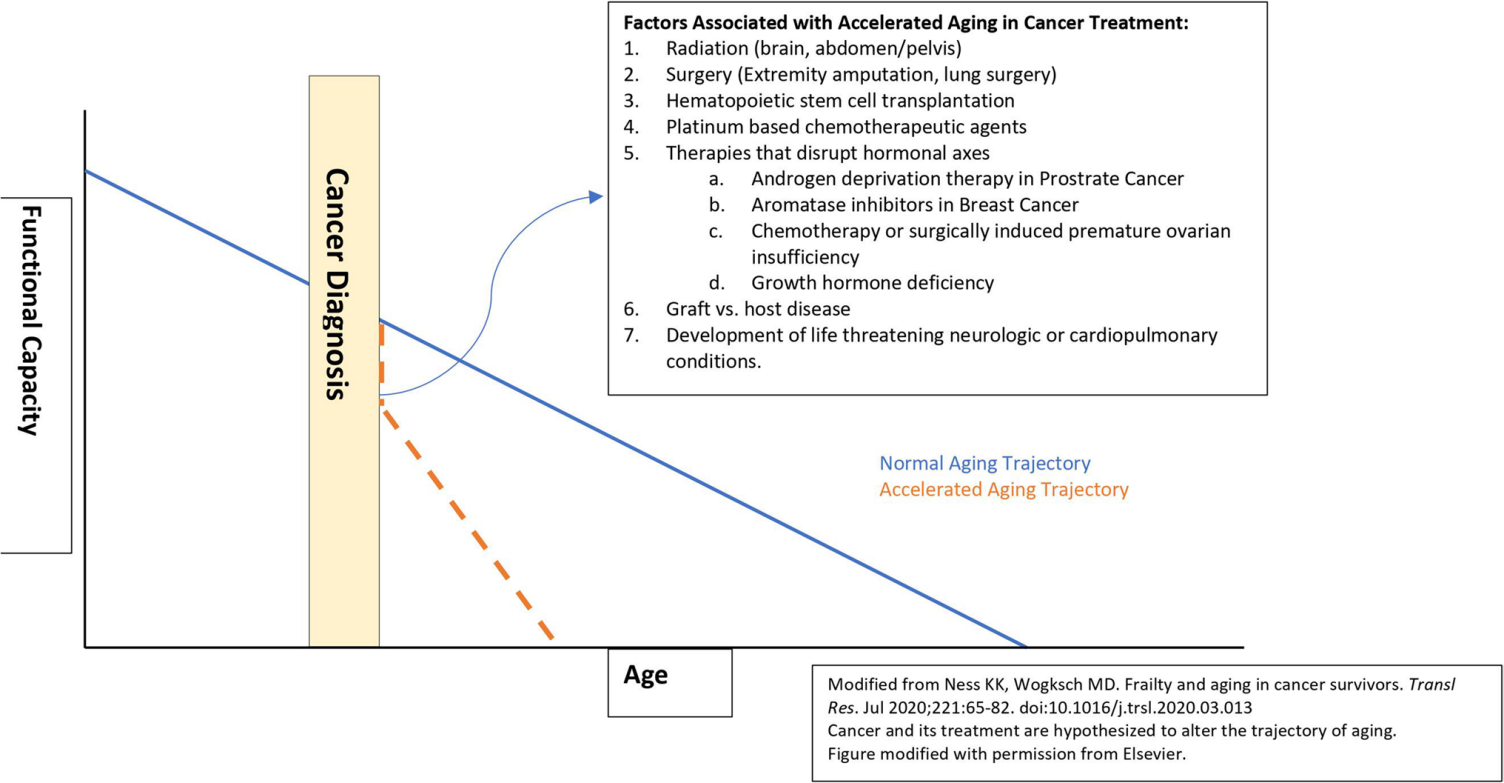
Normal aging versus accelerated aging

Over the last 10 years, treatments for hematologic and solid tumor malignancies have been revolutionized through targeted therapies and immunotherapy. However, despite continued efforts to focus treatment in a manner that spares healthy cells and tissues, damage can still occur, contributing to the aging phenotype [13•]. The mechanisms by which this can happen are described in detail below and summarized in Fig. 1.

1. Cellular senescence: defined as the stable arrest of the cell cycle. In addition to apoptosis, cellular senescence inhibits unchecked growth of cells. Senescent cells have a different “secretome” as described in Table 1 compared to non-senescent cells that mirror inflammatory responses and are crucial for the induction and maintenance of senescence. Its impact on cancer development is twofold: on one hand, this inflammatory cascade can prevent pre-malignant cells from realizing their full malignant potential by promoting its clearance. On the other hand, inflammatory cytokines and chemokines can also provide a cancer-promoting microenvironment for neighboring pre-malignant and malignant epithelial cells. Thus, the overall impact depends on the specific tissue, its genome, and external stimuli [14].
  Chemotherapy, CDK4/6 inhibitors, and immunotherapy aim to induce senescence in tumor cells, but they can also exert a similar cellular senescence in adjacent non-tumor tissues [15]. The associated inflammatory cascade associated with senescent cells in tumor-adjacent cells can itself lead to accelerated aging [16, 17].
2. Telomere attrition: telomerase is an enzyme which is associated with telomere lengthening and thereby cellular propagation. Inhibition of telomerase and the resultant short telomeres prevent uncontrolled cell proliferation. When telomere length reaches a critical size, DNA damage occurs, and replicative senescence is achieved. Cancer cells possess high telomerase activity and they are characterized by their infinite ability to replicate [18]. Cancer treatments such as azidothymidine (AZT) and tamoxifen can directly impair telomerase [19]. Indirect effects of chemotherapy on shortening telomeres have also been described in patients with hematologic and solid tumor malignancies, thus accelerating aging in cancer survivors [20].
3. Genomic instability: defects in DNA surveillance mechanisms (DNA repair machinery, DNA damage checkpoint) can result in genomic instability, thus predisposing the cell to malignant transformation.
  Radiation therapy can cause direct DNA damage [21]. In addition, chemotherapeutic agents such as cisplatin can generate reactive oxygen species as intermediates which can also cause DNA damage [22].
4. Epigenetic alterations: epigenetic phenomena such as histone modifications, DNA methylation, and microRNAs can influence the formation of cancer in multiple ways: (i) it can induce point mutations which can cause changes in the function of genes regulating the cell cycle; (ii) it can cause genomic instability, leading to chromosomal breaks, allelic loss, and translocations which is characteristic of many malignancies; and (iii) it can activate/deactivate gene transcription for example of proto-oncogenes and tumor suppressor genes.
  Epigenetic age acceleration (EAA) is the deviation between the estimated epigenetic age and chronological age and represents the general health status or rate of physiologic aging. To ascertain such physiologic aging, several DNA-based “epigenetic clocks” have been proposed and demonstrated strong associations with various age-related chronic health diseases [23].
  Studies of childhood cancer survivors show that they have a higher annual change in EAA compared to non-cancer controls, and this accelerated change persists throughout their lifetimes [24••]. Furthermore, exposure to various cancer therapies can contribute to the heightened EAAs in cancer survivors. For example, alkylating agents, topoisomerase inhibitors, doxorubicin, methotrexate, and radiation therapy can lead to DNA hypermethylation and lead to accelerated aging phenotype [24••, 25]. The mechanism for this phenomenon remains poorly understood.
5. Loss of proteostasis: due to a loss of ability to maintain correctly folded proteins, and degrade nonfunctional or unnecessary proteins, loss of proteostasis can lead to increased cellular apoptosis and is a mechanism leveraged by some cancer therapeutics such as ixazomib which is used in the treatment for multiple myeloma [13•].
6. Deregulated nutrient sensing: everolimus is a mTOR inhibitor with multiple indications including in breast cancer, renal cell carcinoma, and neuroendocrine tumors. However, its use is also associated with the aging phenotype in mice models due to impaired wound healing, insulin resistance, cataracts, and testicular degeneration [26].
7. Altered intercellular communication: immunotherapy such as PD-1 blockade which has ever-broadening indications can stimulate intercellular communication, and is associated with a proinflammatory phenotype associated with aging [13•].

### Indirect Impacts of Cancer and Its Therapy

Cancer patients experience worse quality of life, lost productivity, and increased psychosocial distress and anxiety compared to non-cancer controls [27••, 28]. Furthermore, heightened levels of psychosocial stress are linked to the activation of the sympathetic nervous system and the hypothalamic–pituitary–adrenal (HPA) axis which mediate the release of downstream catecholamines and cortisol. Higher circulating levels of these stress hormones increase the risk of biological aging in three ways: (i) increased cellular metabolic activity, which increases the production of reactive oxygen species which can cause genomic instability as previously described; (ii) cellular damage, or the ability of glucocorticoids to decrease telomerase activity, leading to cellular senescence; and (iii) glucocorticoids are thought to impair DNA repair pathways. This damage can drive inflammatory changes in the nearby tissue microenvironment that is characteristic of senescent cells. Furthermore, stress hormones themselves are associated with an increase in the production of proinflammatory cytokines by leading to the upregulation of inflammation-promoting transcription factors such as NF-KB, AP1, and CREB. This cumulative upregulation in inflammation can result in accelerated aging [29•].

Cancer patients may also develop malnutrition due to adverse effects from chemotherapy and/or radiation due to altered sense of taste and smell, food aversions, nausea/vomiting, mucositis, constipation, diarrhea, and early satiety, consequently leading to weight loss. In one retrospective study of 191 cancer patients aged 28–72 years old, weight loss was observed in 40–90% of cancer patients receiving chemotherapy (depending on cancer location) [30]. In addition, chemotherapy has also been found to have a direct effect on muscle loss independent of weight. For example, cisplatin, irinotecan, doxorubicin, and etoposide cause direct muscle loss by activating transcription factors such as NF-KB and tumor growth factor which upregulate proteolysis and inflammatory cytokines, accelerating muscle catabolism [31].

Malnutrition is associated with early treatment discontinuation and heightened risk of chemotherapy toxicity, which can lead to accelerated aging as described above [32].

In one study of 114 patients with metastatic colorectal cancer, malnutrition was associated with more adverse effects after chemotherapy, especially hematologic and gastrointestinal side effects [33]. This may be partially explained by the dysbiosis of the gut microbiome, a dynamic endocrine entity that is highly receptive to changes in diet and other lifestyle factors. Chemotherapy has been shown to alter the microbiome, including reducing the diversity of microbes, which has been associated with intestinal inflammation and characteristically found in obese or elderly patients [34]. However, the gut microbiome also regulates the metabolism of immunotherapy and many common chemotherapies (cisplatin, oxaliplatin, doxorubicin, cyclophosphamide), including the development of treatment-related toxicity [35]. Therefore, it can be imagined that the development of malnutrition (likely multifactorial) may also lead to accelerated aging due to dysbiosis and which can further lead to treatment-associated toxicity (which thereby leads to a cycle of accelerated aging). On the other hand, modulation of the gut microbiome by the use of probiotics has been associated with the prevention of treatment-related toxicity in colorectal cancer [36].

Unfortunately, the impact of chemoradiotherapy on nutrition may not subside after the completion of treatment. Some studies suggest that chemotherapy and radiation treatments may also be associated with long-term sequelae leading to poor nutritional status. In 40 patients with stage III-IV head and neck cancer assessed at a median follow-up of 44 months post-chemoradiotherapy, 75% experienced dysphagia, and 19% were at risk of malnutrition. All the participants were forced to implement dietary restrictions. Furthermore, their intake of a balanced diet of vitamins and minerals was found to be far below recommended levels [37].

Although a direct mechanism remains to be clearly elucidated, by leading to weight loss, sarcopenia, and long-term nutritional deficits, cancer treatments such as chemoradiotherapy certainly appear to add fuel to the fire that is aging.

## Clinical Manifestations of Accelerated Aging

### Frailty

Frailty is a state of reduced physiologic reserve which occurs as a result of normal aging. Clinically, frailty is used to identify cancer patients at high risk for poor outcomes, and after cancer therapy to identify survivors at risk for early morbidity and mortality, also known as Fried’s criteria [38]. However, there is no standard instrument to identify frailty; rather, its assessment spans multiple domains including physical performance (as delineated by performance on the 6-min walk test, get up and go test, gait speed), strength (grip strength), nutritional status, and cognition and is characterized by an accumulating deficit approach (e.g., totaling up the number of tasks or activities that a patient is not able to do). Furthermore, as it is a dynamic process, patients may move in and out of frail states throughout their lifetimes.

Between 9 and 59% of cancer survivors have been classified as frail in epidemiologic studies—nearly quadruple the rates of frailty compared to non-cancer survivors in analyses matched by age, sex, and race [39–42]. Female cancer survivors are more likely to be frail [43], which may be partially attributable to their capacity to regenerate muscles [44]. Moreover, cancer treatments acting on hormonal axes (e.g., androgen deprivation therapy in prostate cancer, estrogen antagonists such as aromatase inhibitors) may exacerbate frailty [45]. Cancer treatment that involves operative procedures can initiate a cascade of cytokine releases including IL-6 and C-reactive protein (CRP) which may induce proinflammatory changes conducive to aging [46].

Among childhood cancer survivors, those with malignancies involving the brain, bone, or Hodgkin’s lymphoma were at the most risk of developing frailty [43, 47]. Cancer treatment–associated predictors of frailty in survivors are listed in Fig. 1.

#### Identifying Cancer Patients Most at Risk of Developing Frailty

*P16ink4a* is a biomarker whose expression increases with chronologic age, and it may serve as a robust biomarker of molecular aging [48]. In studies of patients receiving chemotherapy, *p16ink4a* expression increased exponentially after treatment and remained elevated at 12 months post-treatment [49]. Some have considered this increase to represent a 10–15-year increase in chronologic age [50], and measuring levels of this biomarker pre- and post-treatment may help oncologists predict which patients may develop treatment-associated frailty. For example, Smitherman et al. [51] found that among children and young adult cancer survivors, chemotherapy was associated with higher p16ink4a levels. Frail survivors exhibited higher levels of p16ink4a levels compared to robust survivors; this higher expression of p16ink4a was found to represent an average 25-year age acceleration in frail survivors [51].

Interleukin (IL) 6 has been a well-studied inflammatory biomarker shown to be correlated with frailty [52], and has been shown to predict functional decline including the capacity to carry out activities of daily living and development of poor mobility [53].

### Inflammaging/Metaflammation

Aside from cancer therapy–related changes in left ventricular ejection fraction (for example, due to anthracyclines), cancer survivors living at least 5 years beyond diagnosis face up to a threefold higher risk of cardiovascular-specific mortality and up to an 18-fold increase in risk factors for cardiovascular disease such as hypertension, diabetes mellitus, and dyslipidemia compared to age-matched counterparts without a history of cancer [54, 55]. This is likely a result of both age coupled with direct (chemotherapy, radiation, targeted therapy) and indirect (frailty, weight gain) effects of cancer therapy [56, 57].

Indeed, studies of cardiorespiratory fitness (CRF), an integrative assessment of global cardiovascular function, demonstrate cancer treatment–associated decline and reduced recovery after treatment [58, 59]. To illustrate, patients with breast cancer have 30–32% lower mean CRF levels compared to age-matched, healthy, *sedentary*, control subjects [60]. Similar phenomena have been illustrated in young adult cancer survivors [61], and in patients with gynecologic cancers [59]. In fact, among cancer survivors, peak exercise capacity (V02 peak) is reduced by an average of 20% at mean follow-up after 34 months of chemotherapy [62]. Furthermore, cancer survivors have been shown to have low physical activity levels, with up to 75% of survivors not achieving recommended guidelines [63]. Yet as few as 20% of oncologists routinely discuss lifestyle guidance in survivorship [64]. Thus, chronic low physical activity, coupled with increasing chronologic age, coupled with late effects of cancer treatment can all compound to lower CRF.

However, undergoing aerobic and resistance training in cancer survivors has been associated with improvement in CRF. Among 152 breast cancer survivors (most of whom had completed therapy), participation in a once-a-week, supervised exercise regimen focused on aerobic and resistance training resulted in significant improvement in CRF, quality of life, and fatigue [65]. In another study of cancer survivors, participation in aerobic and resistance training was associated with improved 6-min walk duration, 1-repetition maximum leg press, and arm strength [66].

As a result, the American Society of Clinical Oncology (ASCO) has issued a practice guideline to better identify cancer survivors who may benefit from cardiac rehab to reduce their risk of cardiac dysfunction. The following patients should be considered for high risk of cardiac dysfunction and thus meriting a referral for cardiac rehab [67]: (1) treatment with high-dose anthracycline or high-dose radiotherapy when the heart is in the treatment field or lower-dose anthracycline in combination with lower-dose radiotherapy; OR (2) treatment with lower-dose anthracycline or trastuzumab alone plus the presence of ≥ 2 risk factors (smoking, hypertension, diabetes mellitus, obesity, dyslipidemia), ≥ 60 years at cancer treatment, or compromised cardiac function; OR (3) treatment with lower-dose anthracycline followed by trastuzumab.

### Can Accelerated Aging Be Reversed or Decelerated?

Given that a higher number of cancer survivors are living longer and aging into older adulthood, evidence-based strategies to mitigate the aging consequences of cancer and cancer treatment described above are desperately needed. However, there are limited data on how physical activity and dietary strategies may avert or ameliorate accelerated aging associated with cancer or its treatment. Furthermore, how diet and exercise may impact biomarkers associated with aging remains to be investigated [27••].

#### The Role of Diet

Animal models suggest that caloric restriction can slow biological aging and can not only delay the onset of cancer but also can delay the onset of cardiovascular disease, diabetes, and neurodegenerative disorders and prolong overall lifespan [68, 69]. Furthermore, during the last decades, diet and nutrition have been implicated in cancer risk, progression, and treatment response through acting on shared aging pathways described above including telomerase activity, bioenergetics, DNA repair, and oxidative stress [70]. To illustrate, it is estimated that 30–40% of cancers can be prevented by lifestyle choices including a healthy diet, improved physical activity, and prevention of obesity and overweight. Studies of immigrants suggest that migration is associated with altered cancer risk compared to the country of origin. For example, first-generation Japanese women in the USA have a threefold increase in breast cancer compared to women living in Japan [71]. Similarly, studies of European migrants to Australia showed a higher risk of colorectal cancer (similar to Australian-born population) but a diminished risk of stomach cancer (similar to Australian-born population), proportional to the migrants’ duration in the country [72]. It is thought that these alterations in cancer risk are at least partially due to dietary acculturation.

Furthermore, calorie restriction (CR), or reduction in dietary intake by 30% without causing malnutrition, has been postulated to curb excess adiposity and thus decrease the level of oxidative stress that promotes tumorigenesis [73]. In vitro studies suggest that CR can not only halt tumor progression but also increase lifespan in experimental animal models [74]. In a prostate cancer mouse model, calorie restriction decreased tumor weight and plasma insulin levels as well as decreased IGF-1 signaling which was correlated with higher apoptosis levels [75]. In human studies, 15% caloric reduction over 4 years showed a decrease in the growth factors cascade associated with increased risk of cancer [74]. Another study of calorie restriction over a 2-year period in humans demonstrated a reduction in markers of oxidative stress associated with tumorigenesis [76].

Thus, diet and nutrition can alter both cancer and aging outcomes. Yet simply recommending dieting and/or fasting can be challenging in patients with malignancies due to the occurrence of sarcopenia and sarcopenic obesity associated with both cancer and its treatment [77]. Additionally, the relationship between diet, nutrition, aging, and the long-term effects of cancer and cancer treatment is not well understood.

Yet, there is evidence to suggest that diet modulation before cancer treatment may help reduce treatment-associated toxicity, improve treatment specificity for the tumor, and reduce treatment effect on non-cancerous healthy tissue, thereby improving survival [78–81]. For example, in a randomized trial of patients with gynecologic cancer, patients who fasted 36 h prior to chemotherapy and 24 h post-chemotherapy demonstrated better chemotherapy tolerance, higher quality of life, and ability to participate in their activities of daily living, and less fatigue than control patients [82].

Furthermore, in particular, whey protein supplementation during cancer treatment has been associated with improved physical performance in cancer patients [83–85]. In one randomized double-controlled study, 40 mg of whey protein supplementation per week for 12 weeks between chemotherapy cycles for patients with non-metastatic solid tumor malignancies led to statistically significant increases in serum albumin levels compared to control, which are reflective of general nutritional status [83]. In another randomized study of malnourished patients (> 10% unintentional weight loss over 6 months prior to the study) with a mix of advanced solid tumor malignancies, 20 mg of whey protein daily for 3 months led to statistically significant increases in muscle strength, body weight, and decreased risk of chemotherapy toxicity compared to the control arm [85].

The mechanism may be through improvement in lean body mass, sarcopenia, muscle strength, and functional capacity thereby attenuating frailty and preventing chemotherapy toxicity. However, there is also evidence of increased IGF-related signaling associated with an increase in protein intake in mouse models that could on the contrary increase tumor growth by inhibiting apoptosis which must be weighed against any positive supplementation effects, but this effect has not been observed in human studies and is of unknown significance [86].

#### The Role of Exercise

In addition to being safe and cost-effective, prior studies indicate exercise can mitigate several hallmarks of aging [87] and can prevent diet-induced cellular senescence [88]. Furthermore, studies of exercise “prehabilitation” (e.g., exercise before cancer surgery or adjuvant treatment) demonstrates that it can not only improve cardiorespiratory fitness [89], but can also lower post-operative complications [90], and support recovery post-treatment, thereby attenuating a frail state. Furthermore, exercise during and after cancer treatment is associated with a reduction in functional decline because of improved cardiovascular health and maintenance of lean muscle mass [91, 92].

Moreover, it may also modulate tumor biology to increase delivery to the receptiveness of chemotherapy as was seen in patients with pancreatic cancer who participated in a pilot exercise program and went on to demonstrate a differential tumor vascular remodeling which was associated with increased chemotherapy efficacy in the experimental group [91]. In addition to pancreatic cancer, there is evidence of similar exercise-associated tumor remodeling in prostrate [93], breast [94], melanoma [95], and Ewing sarcoma tumors [96]. In the murine model of Ewing Sarcoma, moderate aerobic exercise was associated with increased delivery of chemotherapy to the tumor but not to other organs suggesting that exercise may increase chemotherapy specificity for the tumor and may mitigate the toxicity to non-cancerous tissue thereby reducing the propensity of age-associated changes in healthy tissues as has been described above.

#### Senolytics

One area of ongoing research centers on developing pharmacologic models to selectively reduce senescent cells. Senolysis has gained traction in recent years as a potential method to reverse age-related changes. Among the agents being studied are dasatinib and quercetin, whose senolytic effect is thought to be related to the selective clearance of senescent cells in the bone marrow by induction of apoptosis [97]. In addition, studies of an anti-inflammatory agent, fisetin, are also being conducted in childhood cancer survivors to understand if they can lower frailty and other markers of inflammation including insulin resistance and bone resorption [98].

## Conclusion

In summary, this review summarizes both the direct and indirect mechanisms by which cancer and its treatment can accelerate aging. In essence, cancer treatment including chemotherapy and immunotherapy can induce senescence in adjacent non-malignant cells which can propagate an inflammatory cascade associated with aging. In addition, radiation and chemotherapy can lead to genomic instability due to either direct DNA damage (radiation) or the creation of intermediary reactive oxygen species which can subsequently also induce replicative senescence as cellular DNA damage accumulates. Indirectly, patients with cancer experience an upregulated HPA axis which leads to downstream production of catecholamines and cortisol which can further amplify the risk of accelerated aging due to increased metabolic activity, induction of cellular damage, and impairment of DNA repair pathways. However, modulation of diet and exercise, including caloric restriction, whey protein supplementation, and aerobic exercise are just a few of the interventions being studied to reverse or mitigate the accelerated aging seen in patients with cancer.
